# Core–Shell Structured NiFeSn@NiFe (Oxy)Hydroxide Nanospheres from an Electrochemical Strategy for Electrocatalytic Oxygen Evolution Reaction

**DOI:** 10.1002/advs.201903777

**Published:** 2020-03-28

**Authors:** Mingxing Chen, Shenglin Lu, Xian‐Zhu Fu, Jing‐Li Luo

**Affiliations:** ^1^ College of Materials Science and Engineering Shenzhen University Shenzhen 518060 China; ^2^ Key Laboratory of Optoelectronic Devices and Systems of Ministry of Education and Guangdong Province College of Optoelectronic Engineering Shenzhen University Shenzhen 518060 China; ^3^ Department of Chemical and Materials Engineering University of Alberta Edmonton AB T6G 2G6 Canada

**Keywords:** alloy nanospheres, core–shell structured nanospheres, electrocatalysts, electrocatalytic water oxidation, electrochemical etching, electrodeposition, oxygen evolution reaction

## Abstract

Efficient electrocatalysts for the oxygen evolution reaction (OER) are highly desirable because of the intrinsically sluggish kinetics of OER. Herein, core–shell structured nanospheres of NiFe*_x_*Sn@NiFe (oxy)hydroxide (denoted as NiFe*_x_*Sn‐A) are prepared as active OER catalysts by a facile electrochemical strategy, which includes electrodeposition of NiFe*_x_*Sn alloy nanospheres on carbon cloth (CC) and following anodization. The alloy core of NiFe*_x_*Sn could promote charge transfer, and the amorphous shell of NiFe (oxy)hydroxide is defect‐rich and nanoporous due to the selective electrochemical etching of Sn in alkaline medium. The optimized catalyst of NiFe_0.5_Sn‐A displays a remarkable OER performance with a low overpotential of 260 mV to reach the current density of 10 mA cm^−2^, a small Tafel slope of 50 mV dec^−1^, a high turnover frequency of 0.194 s^−1^ at an overpotential of 300 mV, and a robust durability. Further characterizations indicate that the superior OER performance of the core–shell structured NiFe_0.5_Sn‐A nanospheres might originate from abundant active sites and small charge transfer resistance. This work brings a new perspective to the design and synthesis of core–shell structured nanospheres for electrocatalysis through a facile electrochemical strategy.

## Graphical abstract

Through a facile electrochemical strategy including the process of electrodeposition and anodization, core–shell structured NiFeSn@NiFe (oxy)hydroxide nanospheres could be easily prepared with good conductivity, abundant active sites, high electrochemically active surface area, enhanced oxygen vacancy defects, and controllable chemical composition, which demonstrate high activity and long‐term durability for electrocatalytic water oxidation in alkaline condition.

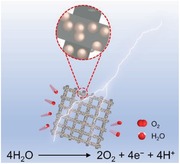

## 1 Introduction

Splitting water into hydrogen and oxygen by renewable energy is attracting enormous attention due to the energy crisis and environmental problems caused by the consumption of fossil fuels.^[^ ^1^, ^2^, ^3^, ^4^, ^5^ ^]^ However, as a half reaction, oxygen evolution reaction (OER) involves multiple electron and proton transfer and is considered to be the bottleneck of the overall water splitting.^[^ ^6^ ^]^ It is of vital importance to develop efficient OER catalysts to accelerate the sluggish reaction and reduce the energy consumption.^[^ ^7^, ^8^, ^9^ ^]^ Unfortunately, although the noble metal‐based catalysts (e.g., IrO_2_, RuO_2_) have been recognized to be the state‐of‐the‐art OER catalysts, their dearth and high cost greatly impede their scalable application and commercialization. Therefore, developing OER catalysts with low cost as well as high performance is still a great challenge.

Earth‐abundant transition metal (especially Fe, Co, Ni) oxides or hydroxides, which possess multiple oxidation states, are often regarded as alternatives to precious metal‐based catalysts towards OER.^[^ ^10^, ^11^ ^]^ Among them, NiFe (oxy)hydroxide has been proven to be one of the most effective materials under alkaline conditions. It is worth noting that transition metal oxides or hydroxides are semiconductors with poor intrinsic conductivity, which greatly obstruct their catalytic performance.^[^ ^12^ ^]^ Thus, different strategies are developed to enhance electron transfer. One common approach is to fabricate metallic precursors (metals,^[^ ^13^ ^]^ alloys,^[^ ^14^, ^15^ ^]^ phosphides,^[^ ^16^, ^17^, ^18^ ^]^ nitrides,^[^ ^19^, ^20^, ^21^ ^]^ borides^[^ ^22^ ^]^) as precatalysts. The surface would be oxidized to oxide or hydroxide as the real active sites, which is evidenced by the experimental observations after the process of OER. The heterojunction between the metallic core and the (oxy)hydroxide shell is of great benefit to charge transport and OER activity.^[^ ^23^, ^24^ ^]^ For example, the improved electron transfer could be attributed to the in situ‐formed interface metal/metal hydroxide heterojunction.^[^ ^23^ ^]^ Coupling with electrically conductive materials (carbon nanotube,^[^ ^25^ ^]^ graphene^[^ ^26^ ^]^) seems to be another promising method. There are also some studies that investigate how the vacancies increase the conductivity of the electrocatalysts.^[^ ^27^, ^28^ ^]^ According to density functional theory calculations, introducing oxygen vacancies led to easy excitation of delocalized electron to conduction band, and hence the conductivity was enhanced.^[^ ^28^ ^]^

On the other hand, the electrocatalytic OER process occurs at the electrode/electrolyte interface, which means that higher value of electrochemical active surface area (ECSA) could provide larger number of active sites and be accessible to more reactants. Various tactics have been developed to increase ECSA, including downsizing the bulk material to nanoparticle (even to single atom),^[^ ^29^ ^]^ exfoliating layered double hydroxide into ultrathin nanosheet,^[^ ^30^ ^]^ removing template,^[^ ^31^, ^32^, ^33^ ^]^ selective etching,^[^ ^34^, ^35^, ^36^, ^37^, ^38^ ^]^ and so forth. Recently, electrochemical etching of the elements (Sn, Al, Zn, etc.) that would generate soluble coordination complexes in alkaline conditions appears to be a facile method to generate nanoporous structure, which also accompanies with the formation of crystal defect (e.g., oxygen vacancy). For instance, the greatly enhanced catalytic activity of perovskite hydroxide CoSn(OH)_6_ after electrochemical etching might originate from the oxygen vacancies and in situ generated nanoporous CoOOH.^[^ ^34^ ^]^ The structural transformation of Zn_0.35_Co_0.65_O to γ‐Co(O)OH with the leaching of zinc would effectively improve the OER activity.^[^ ^35^ ^]^ Besides electrocatalysis, electrochemical etching could also be applied in the fields related to supercapacitors and photocatalysis.^[^ ^39^, ^40^ ^]^

Due to the controlled composition and modulated physicochemical properties, alloys have been widely adopted in many fields.^[^ ^41^, ^42^, ^43^, ^44^, ^45^, ^46^, ^47^, ^48^, ^49^ ^]^ One example is oxygen reduction reaction (ORR).^[^ ^41^, ^42^ ^]^ Precious metal‐based alloys incorporating transition metals as ORR electrocatalysts could not only lower the cost, but also enhance the catalytic activity. However, there are few researches on alloys in the OER system.^[^ ^44^, ^45^, ^50^ ^]^ Furthermore, the preparation of alloy always requires harsh conditions, including but not limited to high temperature, long reaction time, hazardous chemicals, and special experimental techniques (magnetron sputtering,^[^ ^44^ ^]^ arc melting,^[^ ^45^ ^]^ electrospinning,^[^ ^46^ ^]^ etc.). In contrast, electrodeposition has been proven to be a cost‐effective, timesaving, scalable, and universal method in the synthesis of nanomaterials with good reproducibility.^[^ ^51^ ^]^ In addition, the electrodeposited alloy nanospheres can tightly grow on the electrode without the help of any polymer binder. At the same time, it is easy to regulate the chemical composition, ratio, and morphology.

**Figure 1:**
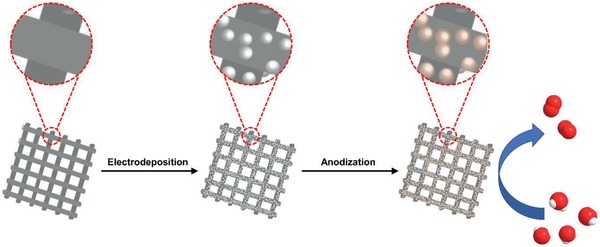
Schematic illustration of the electrochemical strategy to fabricate the core–shell nanospheres and the OER catalytic function.

Herein, we employ a two‐step electrochemical strategy to fabricate the core–shell structured nanospheres as highly active OER electrocatalysts, as shown in **Figure** **1**. The alloy nanospheres of NiFe*_x_*Sn could be prepared by a facile and fast method of electrodeposition. The surface of NiFe*_x_*Sn alloy nanospheres would go through an electro‐oxidation to generate NiFe (oxy)hydroxide amorphous shell, resulting in a core–shell structure. The metallic core of NiFe*_x_*Sn facilitates electron transfer to the shell of amorphous NiFe (oxy)hydroxide, while the shell prevents the further oxidation of metallic core in turn. Selective electrochemical etching of Sn in alkaline solution brings about larger surface area, which could expose abundant active sites and be favorable for mass diffusion and transport. By taking advantage of facile control and regulation of electrodeposition, it is easy to optimize the ratio of Ni/Fe to achieve better OER activity. The resultant core–shell structured nanospheres exhibit high performance and stability towards OER and have the potential for practical application.

## 2 Results and Discussion

**Figure 2:**
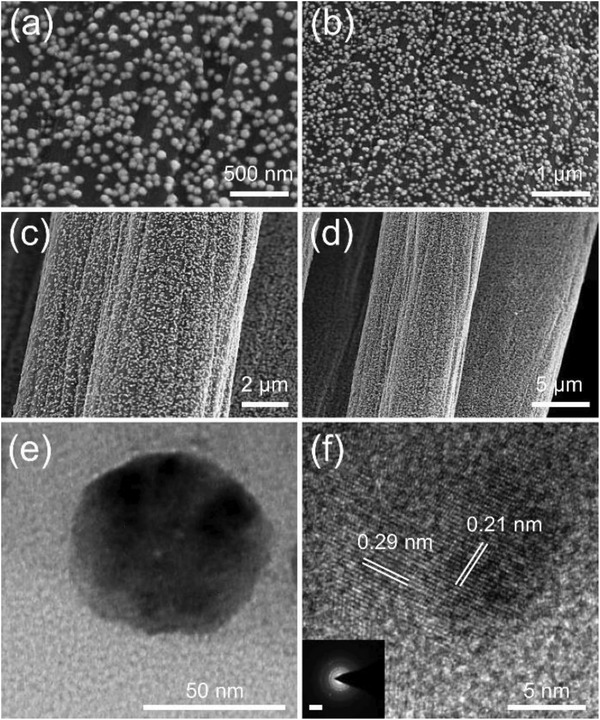
a–d) SEM, e) TEM, and f) HRTEM images of the NiFe_0.5_Sn nanospheres. Inset of (f) shows the SAED pattern with a 5 nm^−1^ scale bar.

The NiFe*_x_*Sn alloy nanospheres are electrosynthesized at −1.2 V versus Ag/AgCl with a total charge of 1 C. The electrolytes contain sodium citrate, tin (IV) chloride and transition metal (Ni, Fe) salts. For example, the NiFe_0.5_Sn alloy nanospheres are grown on a carbon cloth (CC) electrode in the solution where the molar ratio of Ni/Fe is 1:0.5. The Ni/Fe ratio of alloy nanospheres would vary with the transition metal ion concentrations of the electrolytes. The alloys without Fe or Sn are also prepared as control samples (details of the synthesis could be seen in Section 4). The morphology is firstly characterized by scanning electron microscopy (SEM). Uniform NiFe_0.5_Sn nanospheres of about 50 nm in size could be observed on the surface of CC electrode in **Figure** **2**a–d. However, the absence of iron salt in the electrolyte only generates irregular NiSn nanoparticles (Figure S1, Supporting Information). Changing the Ni/Fe ratio to 1:0.1 in the electrolyte results in regular nanoparticles of NiFe_0.1_Sn, as shown in Figure S2, Supporting Information. Meanwhile, the agglomeration is greatly reduced. The nanoparticle would evolve into spherical structure with further increasement of Fe^3+^, however, there are little changes in the morphological feature even when the Ni/Fe ratio reaches 1:1 (NiFe_1_Sn) in the solution (Figure S3, Supporting Information). We also prepare nanospheres without Sn at the Ni/Fe ratio of 1:0.5 in the electrolyte (denoted as NiFe_0.5_). The distribution of NiFe_0.5_ is sparser than those NiFe*_x_*Sn alloys (Figure S4, Supporting Information), even the total applied charge during the electrodeposition of NiFe_0.5_ is 2 C. Thus, Sn might play an important role in the nucleation during the process of electrodeposition.

Transmission electron spectroscopy (TEM) measurements are also carried out to identify the morphology as well as the composition. The NiFe_0.5_Sn nanoparticle is confirmed to be spherical (Figure 2e) and the diameter is 50 nm, which consist with the SEM results. Moreover, the high‐resolution TEM (HRTEM) image (Figure 2f) and the selected area electron diffraction (SAED) pattern (inset of Figure 2f) demonstrate the polycrystalline nature of NiFe_0.5_Sn, while the lattice spacings of 0.29 and 0.21 nm could be indexed to the (101) and (102) plane, respectively. The TEM images of NiSn take the shape of irregular nanoparticles (Figure S5a, Supporting Information), and the lattice fringes have similar interplanar distances of 0.29 and 0.21 nm. The spherical NiFe_0.5_ nanoparticle with a lattice spacing of 0.21 nm is observed in Figure S6, Supporting Information. The elements of Ni, Fe, Sn could be found in the energy‐dispersive X‐ray spectroscopy (EDX) analysis of NiFe_0.5_Sn (Figure S7, Supporting Information), while the signal for Fe and Sn has disappeared in the EDX spectra of NiSn and NiFe_0.5_, respectively (Figures S8 and S9, Supporting Information). The other peaks of Cu and C are derived from the carbon‐coated copper grid.

**Figure 3:**
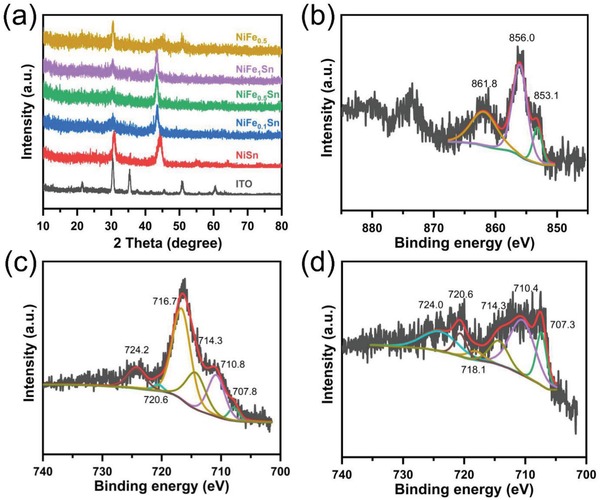
a) XRD patterns of blank ITO, NiFe*_x_*Sn, and NiFe_0.5_ alloys. b) Ni 2p and c) Fe 2p XPS spectra of NiFe_0.5_Sn. d) Fe 2p XPS spectrum of NiFe_0.5_.

The structural characteristics are examined by grazing incidence X‐ray diffraction (GIXRD), as shown in **Figure** **3**a. Herein, the substrate is a flatten indium tin oxide (ITO) electrode rather than a CC electrode. For comparison, the XRD pattern of a blank ITO is also displayed in Figure 3a. In the XRD pattern of NiSn, the peaks observed at 30.7°, 55.0°, 63.9°, and 73.4° could be well indexed to the (101), (201), (202), (211) plane of hexagonal NiSn alloy (JCPDS No. 06‐0414), as shown in Figure S10a, Supporting Information. In addition, the broad peak at 44.6° might be an overlapping of two peaks at 43.5° and 44.6°, corresponding to the (102) and (110) plane of NiSn, respectively, which is in line with the HRTEM result. A shoulder peak at 30.4° could be found from the broad peak of NiSn, and it seems to be well‐matched with the blank ITO's peak. The atomic radius of Ni is a little smaller than that of Fe. When the Ni atom is replaced by Fe atom, the crystalline lattice would expanse. Thus, introducing iron into NiSn would lead the XRD patterns to shift to lower angles, which indicates that the atom of iron has been incorporated into the structure of NiSn.^[^ ^52^, ^53^, ^54^ ^]^ The XRD pattern of NiFe_0.5_ shows two main peaks, which might include two kinds of Ni‐Fe alloys (Figure S10b, Supporting Information). The peak at 44.6° is indexed to Kamacite (JCPDS No. 37–0474), while the peaks at 43.5° and 50.7° are the characteristic peaks of Taenite (JCPDS No. 47‐1417). Moreover, the peak at 30.4° is observed in the all XRD patterns of Figure 3a, suggesting that it might be a mixed XRD peaks of ITO and NiFe*_x_*Sn around 30.4°. Thus, the as‐prepared catalysts are proven to be metallic nanosized alloys.

X‐ray photoelectron spectroscopy (XPS) is employed to further probe the surface valance state and chemical composition. The binding energy of C 1s at 284.6 eV is first used to calibrate the XPS spectra. The existence of Ni and Sn is confirmed by the XPS survey spectrum of NiSn (Figure S11a, Supporting Information). The Ni 2p narrow scan spectrum is resolved to explore the information of valence, as shown in Figure S11b, Supporting Information. The peak at 853.0 eV reveals the metallic state of nickel, while the peaks of 854.1 and 856.4 eV are attributed to oxidized nickel due to the surface oxidation of nickel under air.^[^ ^52^, ^54^, ^55^ ^]^ The small peak at 862.0 eV is a satellite peak. Metallic Sn could be identified by the peaks at 485.0 and 493.6 eV in Figure S11c, Supporting Information.^[^ ^56^ ^]^ As for NiFe_0.5_Sn, apart from Ni and Sn, the element of Fe is also found in the XPS survey spectrum (Figure S12a, Supporting Information). In the Ni 2p narrow scan spectrum, the fitted peak at 853.1 eV in Figure 3b could be assigned to the metallic nickel, while the peak of 856.0 eV is ascribed to nickel oxide. The broad peak at 861.8 eV is the corresponding satellite peak. Zero‐valent tin could also be found at 485.1 eV in the narrow scan spectrum of Sn 3d (Figure S12b, Supporting Information). Unfortunately, the Sn 3p binding energy region and the Ni LMM Auger peak overlap with the Fe 2p binding energy region under an Al electron source (Figure S12a, Supporting Information),^[^ ^38^, ^53^, ^55^, ^57^, ^58^, ^59^ ^]^ and thus the XPS analysis of Fe is seriously interfered with, as shown in Figure 3c. The resulted broad peak could be divided into two peaks at 714.3 and 716.7 eV, which belong to Ni LMM Auger peak and the Sn 3p binding energy region, respectively.^[^ ^53^, ^58^ ^]^ However, metallic iron is successfully identified by the peaks at 707.8 and 720.6 eV in the Fe 2p narrow scan spectrum.^[^ ^52^, ^53^, ^54^ ^]^ The two peaks at 710.8 and 724.2 e V represent the iron oxide. In sharp contrast, in the case of catalyst NiFe_0.5_ without Sn, evident peaks of metallic iron at 707.3 and 720.6 eV are observed in the Fe 2p narrow scan spectrum of NiFe_0.5_, as shown in Figure 2d. The other peaks are assigned to iron oxide, Ni LMM Auger peak and satellite peak. Moreover, the vanished peak around 716 eV manifests the absence of Sn, which could be further verified by the XPS survey spectrum (Figure S13a,c, Supporting Information). The peak of metallic nickel is also well resolved in Figure S13b, Supporting Information. Similar results are obtained in the XPS spectra of NiFe_0.1_Sn and NiFe_1_Sn (Figures S14 and S15, Supporting Information), suggesting that the as‐prepared catalysts are metallic alloys, which is in accordance with the results of XRD and TEM.

**Figure 4:**
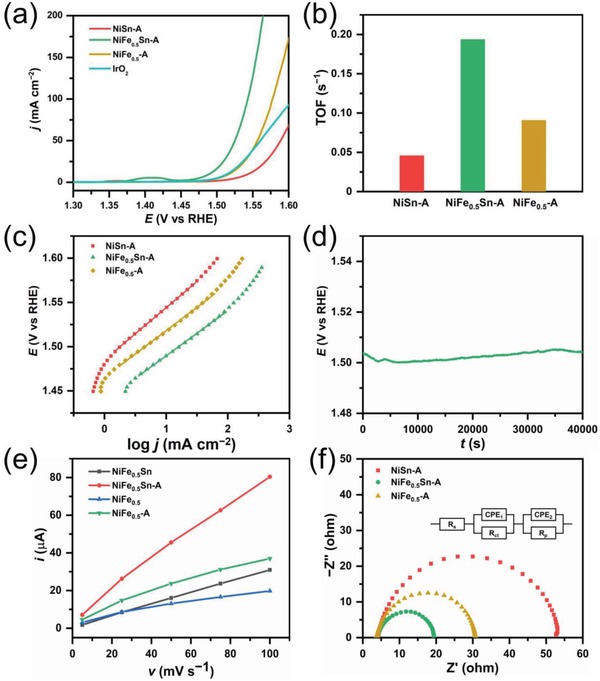
a) Polarization curves of NiSn‐A, NiFe_0.5_Sn‐A, NiFe_0.5_‐A, and Ir/C. b) bar chart for a comparison of TOF of NiSn‐A, NiFe_0.5_Sn‐A, NiFe_0.5_‐A. c) Tafel plots of NiSn‐A, NiFe_0.5_Sn‐A, and NiFe_0.5_‐A. d) chronopotentiometry plot of NiFe_0.5_Sn‐A. e) Cdl calculations of NiFe_0.5_Sn and NiFe_0.5_ before and after anodization. f) Nyquist plots of NiSn‐A, NiFe_0.5_Sn‐A, NiFe_0.5_‐A at 1.53 V. The inset two‐time constant circuit consists of a solution resistance (*R*_s_), two constant phase elements (CPE), a resistance related to surface porosity (*R*_p_) and a charge‐transfer resistance (*R*_ct_).

The electrocatalytic OER performances are evaluated in 1 m KOH using a three‐electrode system at room temperature. The linear sweep voltammogram curves of different alloys are conducted at the scan rate of 5 mV s^−1^ to minimize the capacitive current, as shown in Figure S16a, Supporting Information. The blank CC shows almost no catalytic activity, demonstrating that the enhanced currents are originated from the NiFe*_x_*Sn alloys. Sn‐catalyst without Ni and Fe is also prepared under the same condition, whose performance could rule out the possibility that Sn might be accountable for OER. The electrochemical performance of NiFe_0.5_Sn exceeds all other alloys', which might be due to the most appropriate electronic structure modified by the Ni/Fe ratio.^[^ ^53^ ^]^ For the purpose of surface reconstruction as well as selective etching of Sn, the alloy precatalysts would undergo a 2‐h galvanostatic anodization at a current density of 10 mA cm^−2^ (denoted as NiFe*_x_*Sn‐A). The electrocatalytic activity would be enhanced after anodization. For example, as compared with NiFe_0.5_Sn, the NiFe_0.5_Sn‐A after anodization displays about 12 mV negative shift of potential to reach the same current density (Figure S16b, Supporting Information). It is obvious that introducing Fe would lead to the greatly enhanced electrochemical performance, since NiSn and NiSn‐A without Fe exhibit poor OER performances in **Figure** **4**a and Figure S16a, Supporting Information. In addition, the anodic peaks shift to more positive potentials along with the increasing concentrations of Fe, which agrees with previous works.^[^ ^59^ ^]^ We could find that NiFe_0.5_Sn‐A displays the highest activity among those catalysts. In Figure 4a, the NiFe_0.5_Sn‐A exhibits the lowest onset potential. The onset potentials of commercial IrO_2_ and NiFe_0.5_‐A are similar, which are more negative than that of NiSn‐A. However, the current density of NiFe_0.5_‐A increases sharply after the potential of 1.5 V, which surpasses that of IrO_2_ gradually. As a benchmark for the solar‐to‐fuel system, the overpotential to reach the current density of 10 mA cm^−2^ is always considered to be a metric to evaluate the performance of water splitting. For NiFe_0.5_Sn‐A, it only needs an overpotential of 260 mV to reach the current density of 10 mA cm^−2^. The overpotential of NiFe_0.5_Sn‐A at 10 mA cm^−2^ and its onset overpotential are better than most reported NiFe‐based catalysts (Table S1, Supporting Information). However, the corresponding overpotential value for NiSn‐A, NiFe_0.1_Sn‐A, NiFe_1_Sn‐A, NiFe_0.5_‐A was 310, 295, 270, 287 mV, respectively (Figure 4a and Figure S16c, Supporting Information). Table S2, Supporting Information, shows the element contents of Ni and Fe in the catalyst, as determined by inductively coupled plasma optical emission spectroscopy (ICP‐OES). Thus, the turnover frequency (TOF) is adopted to compare the intrinsic activity. The TOF of NiFe_0.5_Sn‐A at the overpotential of 300 mV is 0.194 s^−1^, which is about 4.2 and 2.1 times higher than that of NiSn‐A and NiFe_0.5_‐A, respectively (Figure 4b). It is important to note that the TOF has been largely underestimated because only a small number of nickel and iron in the catalyst actually catalyze OER. The mass activity of the catalyst has been provided to further access the intrinsic electrochemical performance for OER (Figure S16d, Supporting Information). To achieve 3500 A g^−1^, the NiFe_0.5_Sn‐A only needs the potential of 1.56 V, which is superior to NiSn‐A (1.60 V) and NiFe_0.5_‐A (1.58 V). Tafel analysis is conducted to get more information on the OER kinetics (Figure 4c and Figure S16e, Supporting Information). The Tafel slope of NiFe_0.5_Sn‐A is 50 mV dec^−1^, which is smaller than that of NiSn‐A (59 mV dec^−1^), NiFe_0.1_Sn‐A (57.8 mV dec^−1^), NiFe_1_Sn‐A (51 mV dec^−1^), NiFe_0.5_‐A (52.7 mV dec^−1^), indicating its more rapid reaction rate during the OER. The long‐term durability is examined by the chronopotentiometric curve recorded at the current density of 10 mA cm^−2^, as shown in Figure 4d. The applied potential of NiFe_0.5_Sn‐A remains almost unchanged at 1.5 V during the 40 000 s test, demonstrating its excellent stability. Moreover, the NiFe_0.5_Sn‐A remains stable at larger current densities from multipotential steps (Figure S16f, Supporting Information).

Moreover, the double layer capacitance (*C*_dl_) before and after anodization are calculated based on cyclic voltammetry (CV) in the non‐Faradaic region (Figure S17, Supporting Information). The *C*_dl_ of NiFe_0.5_‐A has increased from 0.17 to 0.3 mF, which might be caused by the generation of amorphous metal oxides or hydroxides on the surface (Figure 4e). By comparison, the *C*_dl_ of NiFe_0.5_Sn‐A increases from 0.3 to 0.75 mF. Considering that ECSA is proportional to *C*_dl_, we could conclude that electrochemical etching of Sn is beneficial for increasement of ECSA. We also investigate the electron transfer kinetics during the OER process by means of electrochemical impedance spectroscopy. The Nyquist plots of different catalysts in Figure 4f are obtained at 1.53 V. It is obvious that the charge transfer impedance of NiFe_0.5_Sn‐A is smaller, as determined from the semicircle at low frequency. In the Nyquist plot, the Warburg impedance presents a straight line at low frequency, indicating that the reaction is under the control of mass transfer. No straight line could be observed in Figures S18–S20, Supporting Information. The absence of Warburg region in the low frequency range of Nyquist plot suggests that the mass transport has little influence on the OER.^[^ ^60^ ^]^ The Nyquist plots take the shape of two semicircles (Figures S18–S20, Supporting Information), which could be well fitted by the equivalent circuit of two‐time constant serial model,^[^ ^61^ ^]^ as shown in the inset of Figure 4f. The low frequency time constant (CPE_1_‐*R*_ct_) is related to the process of charge transfer, the other time constant in the high frequency (CPE_2_‐*R*_p_) might be associated with the surface porosity.^[^ ^62^ ^]^ Figure S21, Supporting Information, shows that *R*_ct_ of catalysts all decrease with the increasement of applied potential, indicating the accelerated charge transfer kinetics under larger overpotentials. Moreover, the *R*_ct_ value of NiFe_0.5_Sn‐A is lower than that of NiFe_0.5_‐A and NiSn‐A, which reflects faster electron transfer at the electrode/electrolyte interface.

**Figure 5:**
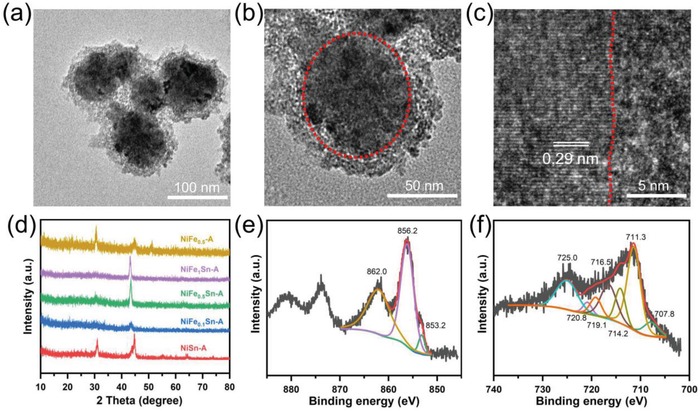
a,b) TEM and c) HRTEM images of SNiFe_0.5_Sn‐A. d) XRD patterns of NiFe*_x_*Sn‐A and NiFe_0.5_‐A, e) Ni 2p, and f) Fe 2p XPS spectra of NiFe_0.5_Sn‐A.

In order to identify the active species for OER, the catalysts after the anodization are also characterized. The morphology in the SEM images does not change evidently, as shown in Figure S22, Supporting Information. However, the TEM images of NiFe_0.5_Sn‐A in **Figure** **5**a,b present a core–shell structure, which is quite different from the TEM result of NiFe_0.5_Sn. The HRTEM image (Figure 5c) reveals that the shell is amorphous and the core is well‐crystallized. The lattice fringe of 0.29 nm in the core, corresponding to the (101) plane, still remains unchanged, while the fluffy shell shows almost no crystal lattice, which could be attributed to the amorphous NiFe (oxy)hydroxide. The elemental mapping analysis of NiFe_0.5_Sn‐A could further demonstrate the alloy@NiFe (oxy)hydroxide core–shell structure, as shown in Figure S23, Supporting Information. In addition, the electron paramagnetic resonance (EPR) measurement is performed to verify the existence of oxygen vacancies (Figure S24, Supporting Information). The NiFe_0.5_Sn‐A shows a stronger signal for oxygen vacancies than NiFe_0.5_‐A does, which suggests that selective etching of Sn is conducive to the formation of oxygen vacancies. Thus, in alkaline media, the NiFe_0.5_Sn alloy is supposed to go through a surface self‐reconstruction process at positive potentials, which includes the generation of NiFe (oxy)hydroxide as well as selective electrochemical etching of Sn. The conjecture is further supported by other characterizations. For example, the XRD pattern of NiFe_0.5_Sn‐A in Figure 5d exhibits a main peak at 43.3°, keeping the same with that of NiFe_0.5_Sn. No other new peak is detected. This result confirms the amorphous nature of the shell and the structural stability of the metallic core. The XPS spectra of NiFe_0.5_Sn‐A are displayed in Figure S25, Supporting Information. The signals of Ni^2+^/Ni^3+^ and Fe^3+^ are presented after peak deconvolution, confirming that the amorphous shell is composed of NiFe (oxy)hydroxide. After Ar^+^ ion etching, the metallic peaks of Ni and Fe are also observed in the Ni and Fe 2p narrow scan spectra, respectively (Figure 5e,f), which are consistent with the results of HRTEM and XRD. Similarly, the core–shell structure of NiFe_0.5_‐A is also obviously affirmed by its TEM images (Figure S26, Supporting Information). The lattice spacing in Figure S26b, Supporting Information, is 0.21 nm, which is essentially identical to that of NiFe_0.5_. In the XPS spectra of NiFe_0.5_‐A, apart from the high valent transition metals in the shell (Figure S27, Supporting Information), zero valent nickel and iron could be found out in the core (Figure S28, Supporting Information). The XPS results of NiSn‐A in Figures S29 and S30, Supporting Information, closely resembled those of NiFe_0.5_‐A and NiFe_0.5_Sn‐A. Moreover, the peaks of metallic core in the XRD patterns of all catalysts show little changes even after the 40 000 s durability test at the current density of 10 mA cm^−2^ (Figure S31, Supporting Information), indicating that the metallic cores are well preserved by the amorphous shells. Taken together, these results suggest that the outstanding electrochemical performance for OER might be attributed to the synergism of the metallic core and the amorphous, defect‐rich shell. The amorphous shell not only functions as the active sites for OER, but also separates the metallic core from the electrolyte, which could prevent the further oxidation of metallic core and ensure the high conductivity, as proved by the TEM, XPS, and XRD results.

## 3 Conclusion

A simple but universal strategy is developed to prepare core–shell structured NiFeSn@NiFe (oxy)hydroxide nanospheres towards OER. The metallic core could improve the electron transfer, while the amorphous shell of NiFe (oxy)hydroxide is responsible for high catalytic activity towards OER and prevents the further oxidation of the metallic core. Meanwhile, the selective etching of Sn is beneficial for enhancing the ECSA as well as creating oxygen vacancies. The low catalyst loading, abundant active sites, rapid charge transfer, and oxygen vacancies are all crucial factors for the high activity of NiFe_0.5_Sn‐A for OER, which can reach the current density of 10 mA cm^−2^ at an overpotential of 260 mV and achieve a TOF of 0.194 s^−1^ at an overpotential of 300 mV. The convenient method to prepare core–shell structured nanospheres might shed light on design and synthesis of catalysts for electrocatalysis in addition to OER.

## 4 Experimental Section

##### Synthesis of the NiFe*_x_*Sn Electrode

In a typical synthesis, the CC (0.5 cm × 2 cm) electrode was ultrasonically washed for at least 30 min in water and alcohol, respectively. Then, a conventional three‐electrode setup was applied to electrodeposit the NiFe*_x_*Sn. The CC electrode was used as working electrode, saturated Ag/AgCl as reference electrode and Pt foil as counter electrode. The NiFe_0.5_Sn alloy nanospheres were prepared through cathodic electrolysis at −1.2 V versus Ag/AgCl in the aqueous electrolyte, which contained Ni(NO_3_)_2_·6H_2_O (0.01 m), Fe(NO_3_)_3_·9H_2_O (0.005 m), SnCl_4_·5H_2_O (0.02 m), and Na_3_C_6_H_5_O_7_·2H_2_O (0.1 m). The electrodeposition would not be finished until the total charge reached 1 C. The alloys of NiSn, NiFe_0.1_Sn, NiFe_1_Sn were electrosynthesized under the same condition, except that the concentration of Fe(NO_3_)_3_ in the electrolyte was 0, 0.001, 0.01 m, respectively. In contrast, there was no SnCl_4_ in the electrolyte of the NiFe_0.5_.

##### Material Characterization

The morphology in SEM image was observed on Hitachi SU‐70 field emission scanning electron microscope with an accelerating voltage of 5 kV. In order to carry out the TEM analysis, the alloy was ultrasonicated from the CC electrode in ethanol, and dropped on the carbon‐coated copper grid. The TEM and EDX results were obtained by a FEI Tecnai G2 F20 transmission electron microscope equipped with Oxford X‐Max 80T. The working voltage was 200 keV. GIXRD patterns of the catalysts were recorded on a Rigaku Smartlab diffractometer with monochromatized Cu Kα radiation (λ = 1.5418 Å), while the grazing incidence was 0.5°. XPS was conducted on a Thermo Scientific K‐Alpha^+^ spectrometer equipped with a monochromatic Al Kα X‐ray source (1486.6 eV) operating at 100 W. Samples were analyzed under vacuum (P < 10^−8^ mbar) with a pass energy of 150 eV (survey scans) or 25 eV (high‐resolution scans). All peaks were calibrated with C1s peak binding energy at 284.6 eV for adventitious carbon. The EPR spectrum was measured by a Bruker Elexsys EPR system at 9.43 GHz. The loading of catalyst was determined by ICP‐OES with an Agilent 720ES spectrometer.

##### Electrochemical Measurements

All electrochemical experiments were performed on a CH Instruments Electrochemical Analyzer (CHI 760E) using a standard three‐electrode system. The CC electrode was adopted as working electrode. The reference electrode was saturated Ag/AgCl, and the counter electrode was Pt foil. The electrocatalytic performances were conducted in 1 m KOH. Linear sweep voltammetry was performed at 5 mV s^−1^ and was corrected for the iR compensation. All potentials in this work were converted to reversible hydrogen electrode (RHE) based on Nernst equation: *E*_RHE_ = *E*_Ag/AgCl_ + 0.059 × pH + 0.197. The process of anodization was performed at the constant current density of 10 mA cm^−2^ for 2 h. The *C*_dl_ could be calculated by the non‐Faradaic cycle voltammetry at different scan rates. The electrochemical impedance experiments were conducted with an amplitude of 5 mV over a frequency range from 0.01 to 100 kHz. TOF values were determined by the following equation: TOF = (*j* × *A*)/(4 × *n* × *F*), where *j* is the current density at a certain overpotential, *A* is the area of the electrode, 4 represents the number of electrons that involve in water oxidation, *n* is the moles of the catalyst and *F* is the Faraday's constant. The ECSA and *C*_dl_ could be calculated according to the following equations: *C*_dl_ = *i*_c_/*v*, ECSA = *C*_dl_/*C*_s_, where *i*_c_ is the charging current, *v* is the scan rate, *C*_dl_ is the double layer capacitance, and *C*_s_ is the specific capacitance value.

## Conflict of Interest

The authors declare no conflict of interest.

## Supporting information

Supporting Information
