# Supplementary material for: Core–Shell Structured NiFeSn@NiFe (Oxy)Hydroxide Nanospheres from an Electrochemical Strategy for Electrocatalytic Oxygen Evolution Reaction
Source: Adv Sci (Weinh). 2020 Mar 28;7(10):1903777. doi: 10.1002/advs.201903777 (PMC7237859; doi:10.1002/advs.201903777)
Supplement: Supplementary file 1 — Supporting Information [file ADVS-7-1903777-s001.pdf]

Copyright WILEY-VCH Verlag GmbH & Co. KGaA, 69469 Weinheim, Germany, 2020.

ADVANCED SCIENCE
Open Access

Supporting Information

for *Adv. Sci.*, DOI: 10.1002/advs.201903777

Core–Shell Structured NiFeSn@NiFe (Oxy)Hydroxide Nanospheres from an Electrochemical Strategy for Electrocatalytic Oxygen Evolution Reaction

Mingxing Chen, Shenglin Lu, Xian-Zhu Fu, and Jing-Li Luo**

Copyright WILEY-VCH Verlag GmbH & Co. KGaA, 69469 Weinheim, Germany, 2020.

Supporting Information

Core-Shell Structured NiFeSn@NiFe (oxy)hydroxide Nanospheres from an Electrochemical Strategy for Electrocatalytic Oxygen Evolution Reaction

Mingxing Chen, Shenglin Lu, Xian-Zhu Fu, Jing-Li Luo**

Dr. Mingxing Chen, Dr. Shenglin Lu, Prof. Xian-Zhu Fu, Prof. Jing-Li Luo
College of Materials Science and Engineering, Shenzhen University, Shenzhen 518060, China
E-mail: (xz.fu@szu.edu.cn, Jingli.Luo@ualberta.ca)

Dr. Mingxing Chen, Dr. Shenglin Lu,
Key Laboratory of Optoelectronic Devices and Systems of Ministry of Education and Guangdong Province, College of Optoelectronic Engineering, Shenzhen University, Shenzhen 518060, China

Prof. Jing-Li Luo
Department of Chemical and Materials Engineering, University of Alberta, Edmonton, Alberta, T6G 2G6, Canada

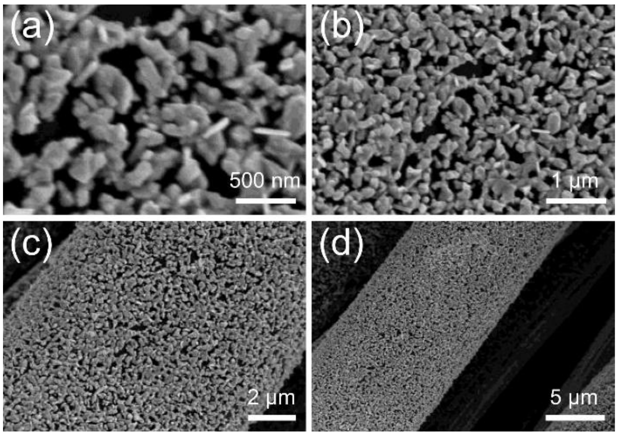

Figure S1. SEM images of NiSn.

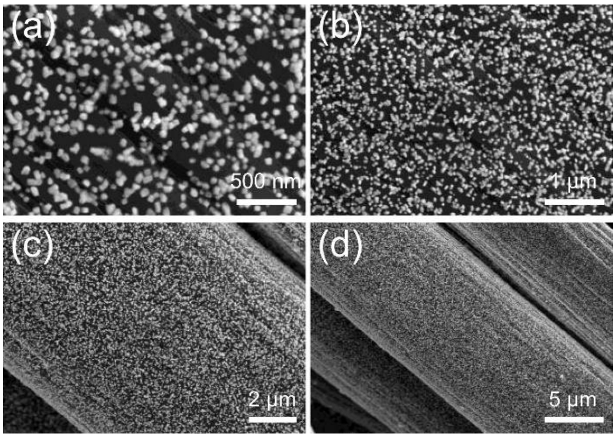

Figure S2. SEM images of $NiFe_{0.1}Sn$.

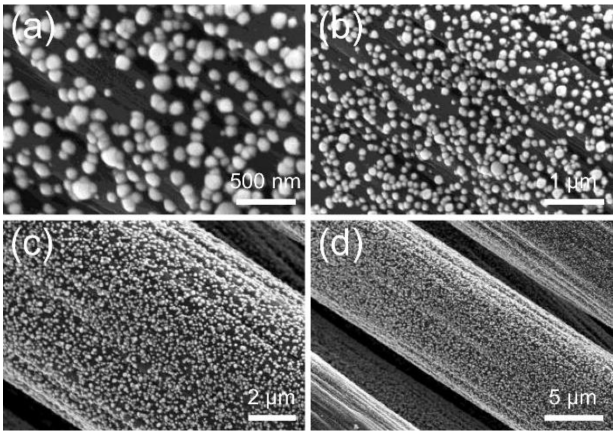

Figure S3. SEM images of $NiFe_1Sn$.

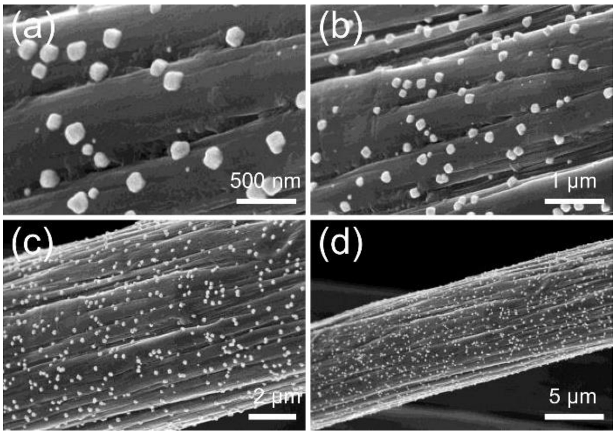

Figure S4. SEM images of $NiFe_{0.5}$.

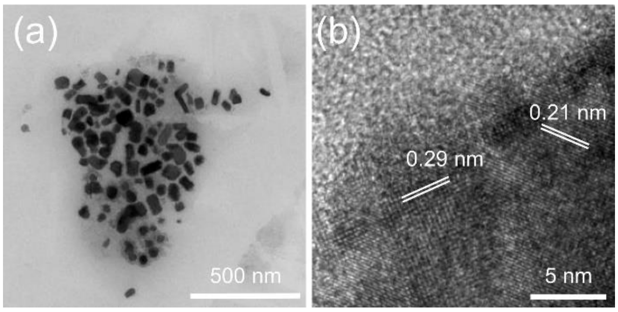

Figure S5. (a) TEM and (b) HRTEM images of NiSn.

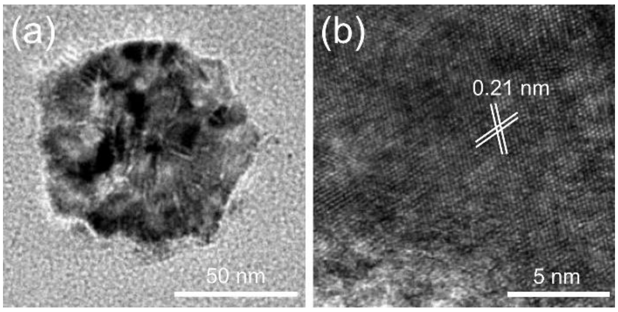

Figure S6. (a) TEM and (b) HRTEM images of $NiFe_{0.5}$.

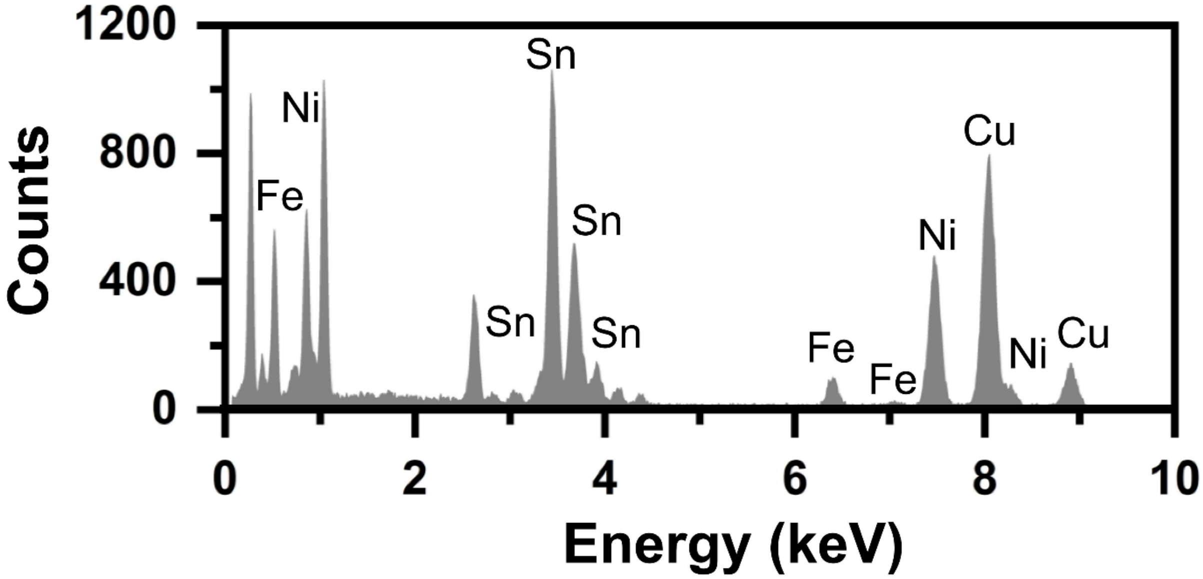

Figure S7. EDX spectrum of $NiFe_{0.5}Sn$.

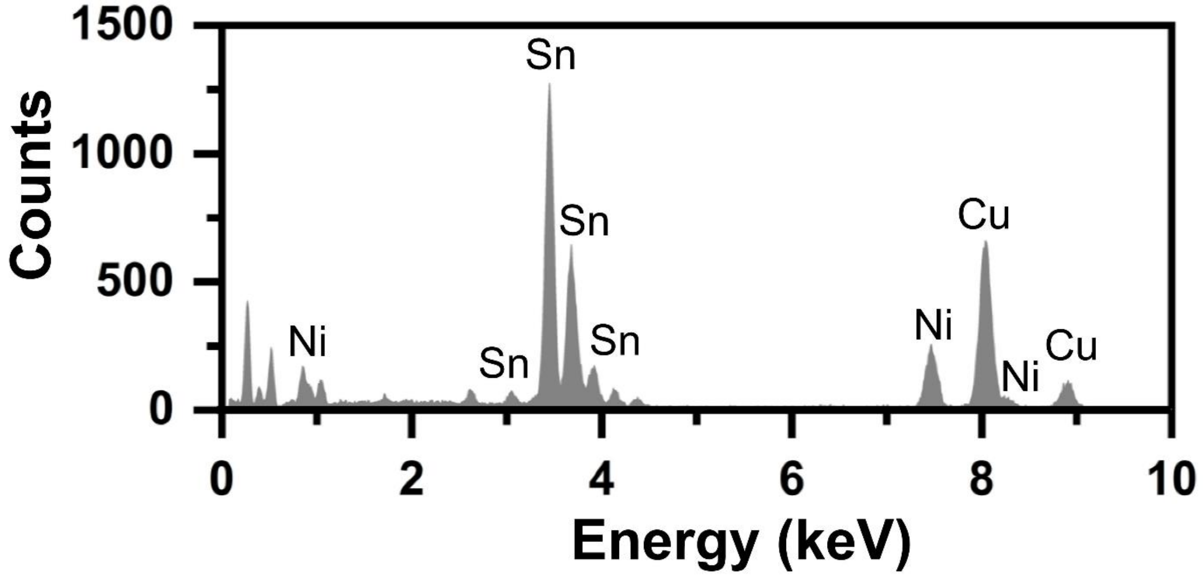

Figure S8. EDX spectrum of NiSn.

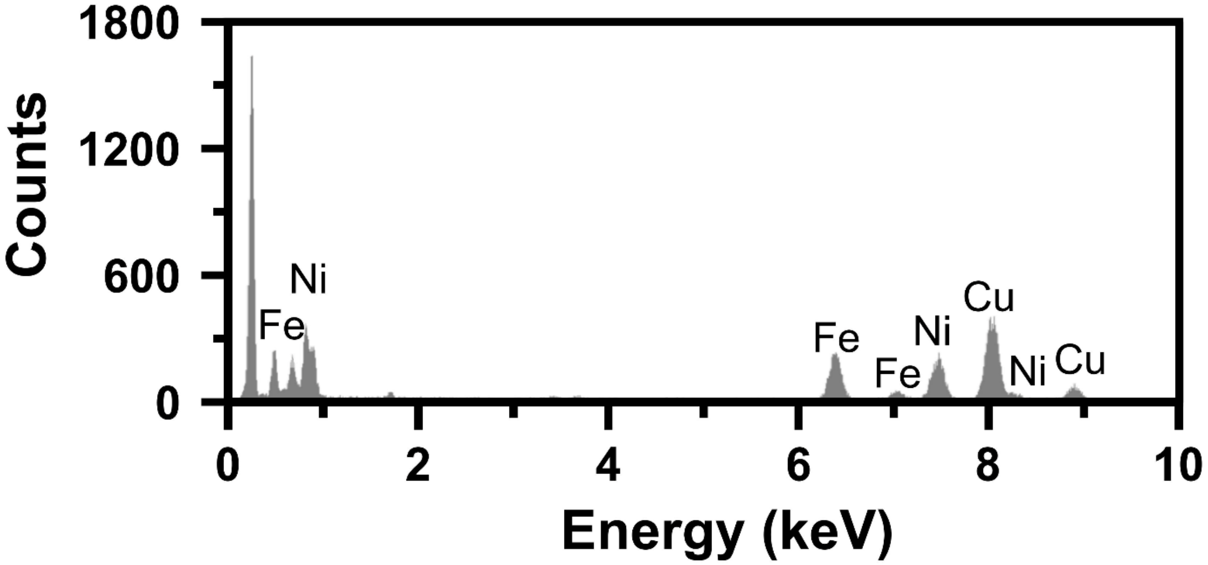

Figure S9. EDX spectrum of $NiFe_{0.5}$.

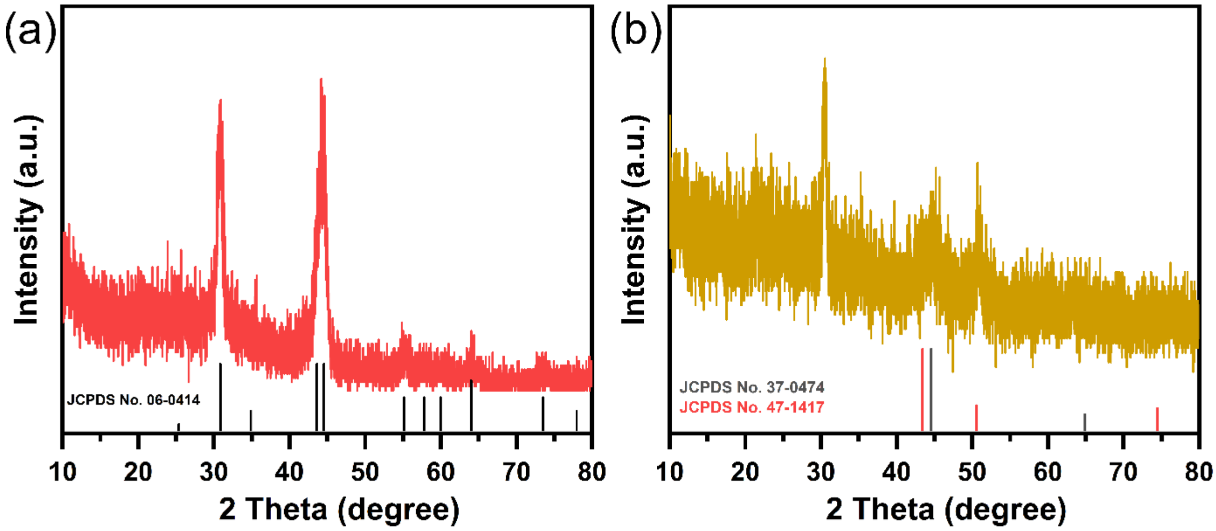

Figure S10. XRD patterns of (a) NiSn and (b) $NiFe_{0.5}$.

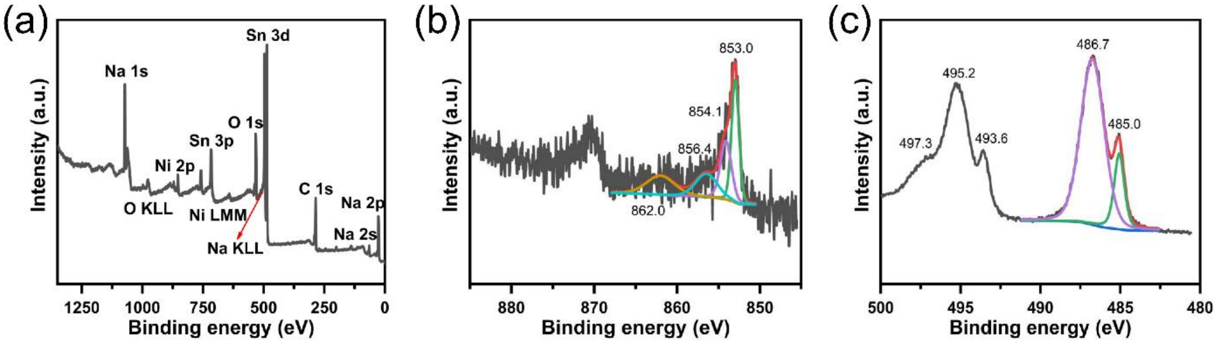

Figure S11. (a) XPS survey, (b) Ni 2p and (c) Sn 3d XPS spectra of NiSn.

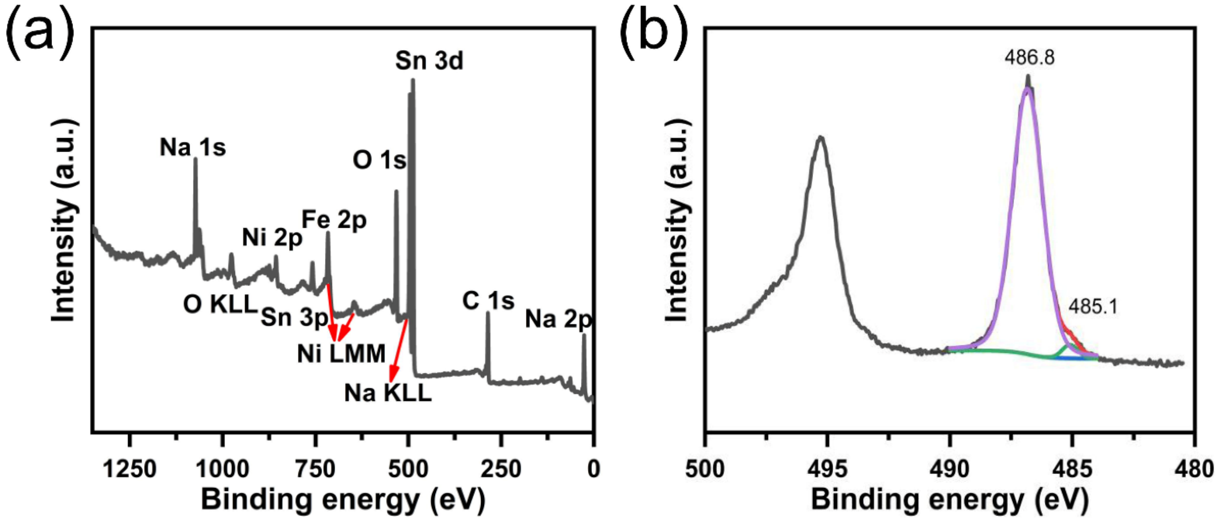

Figure S12. (a) XPS survey, (b) Sn 3d XPS spectrum of $NiFe_{0.5}Sn$.

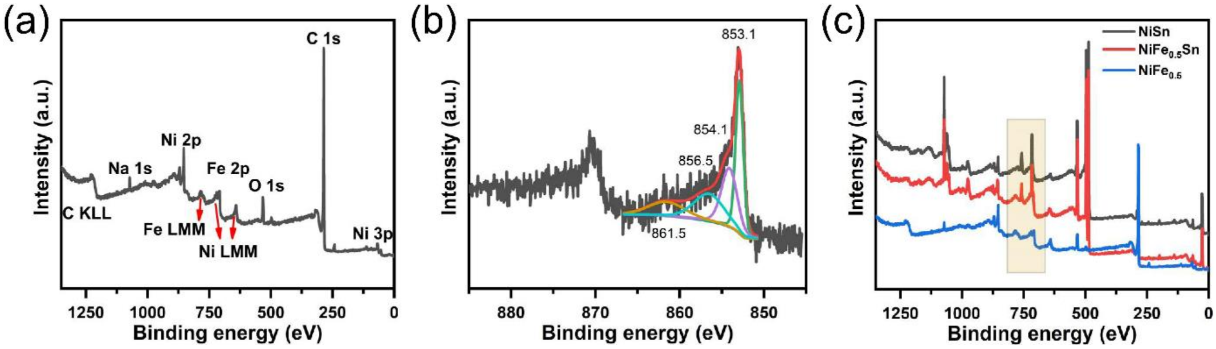

Figure S13. (a) XPS survey, (b) Ni 2p XPS spectrum of $NiFe_{0.5}$, (c) XPS surveys of NiSn, $NiFe_{0.5}Sn$ and $NiFe_{0.5}$.

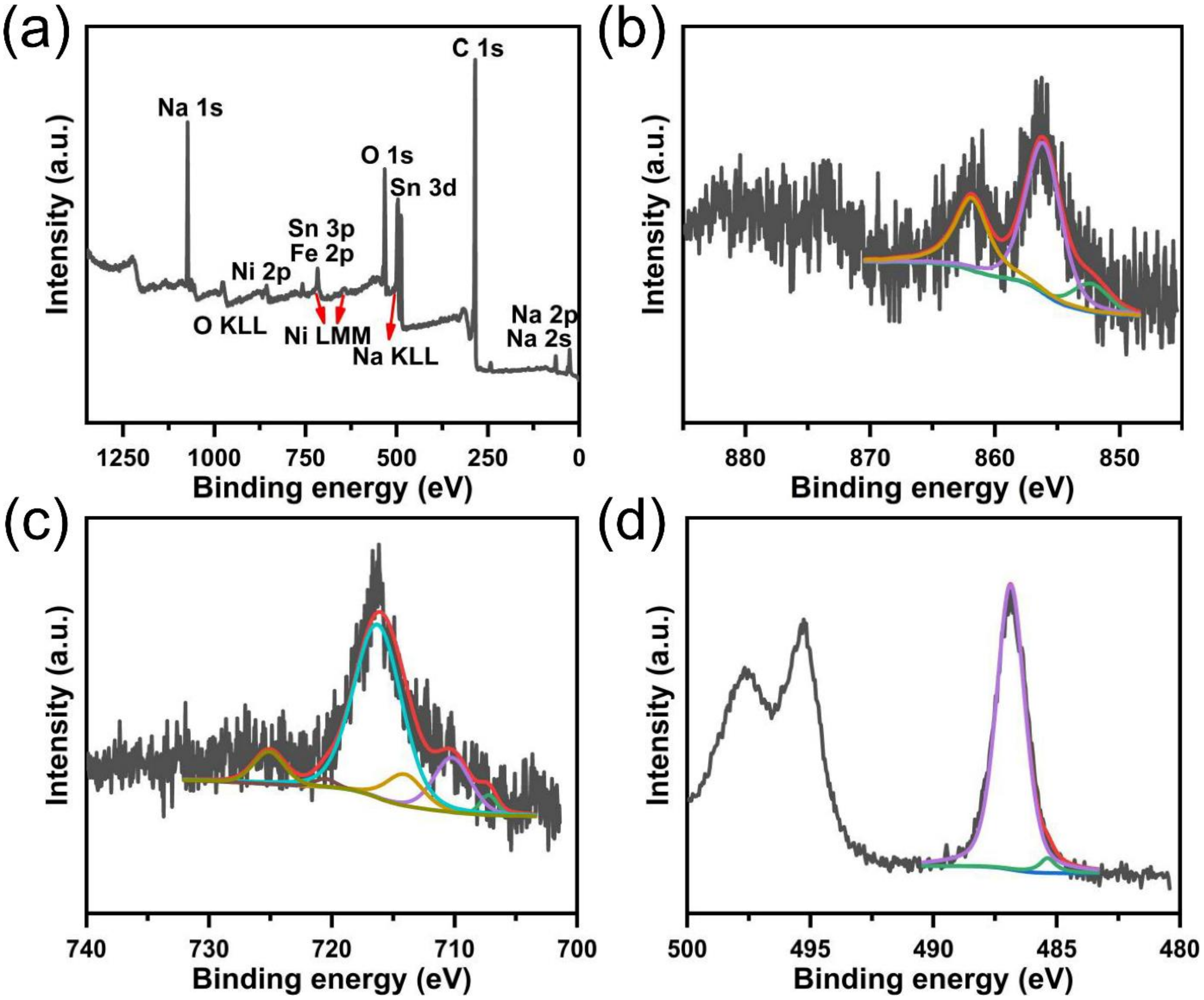

Figure S14. (a) XPS survey, (b) Ni 2p, (c) Fe 2p and (d) Sn 3d XPS spectra of $NiFe_{0.1}Sn$.

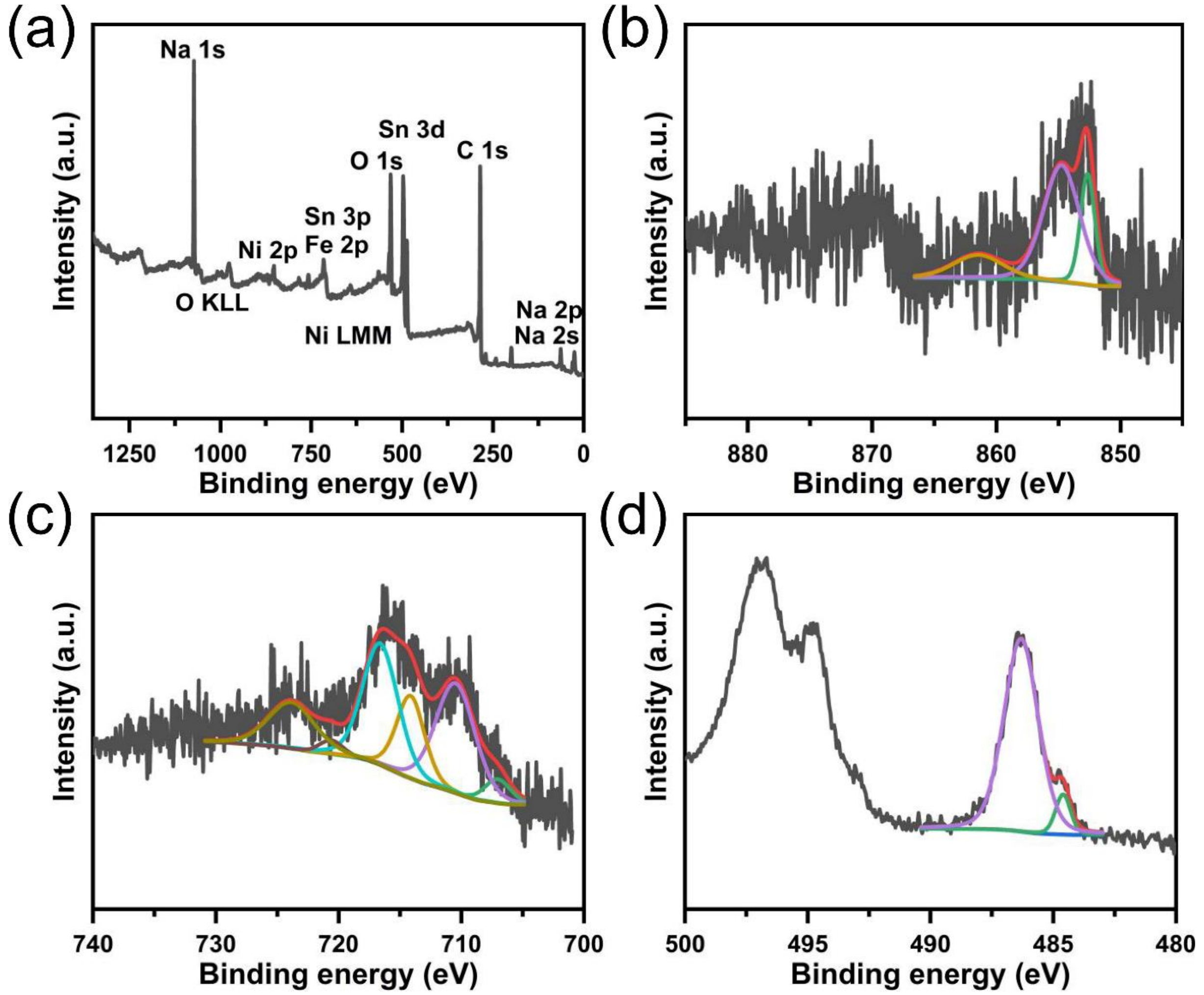

Figure S15. (a) XPS survey, (b) Ni 2p, (c) Fe 2p and (d) Sn 3d XPS spectra of $NiFe_1Sn$.

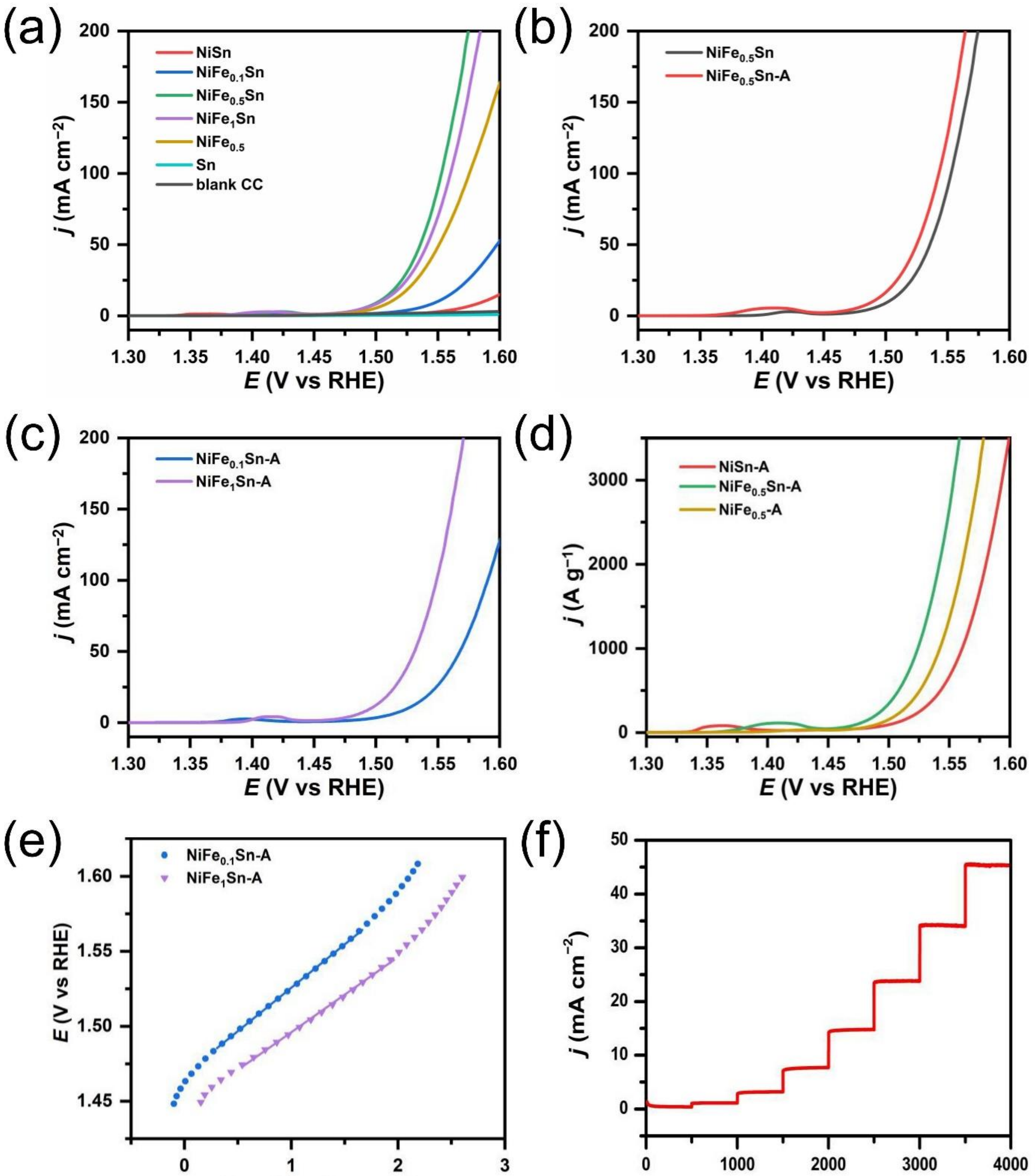

Figure S16. Polarization curves of (a) NiSn, $NiFe_xSn$, $NiFe_{0.5}$, Sn and blank CC, (b) $NiFe_{0.5}Sn$ and $NiFe_{0.5}Sn$-A, (c) $NiFe_{0.1}Sn$-A and $NiFe_1Sn$-A, (d) comparison of mass activity of NiSn-A, $NiFe_{0.5}Sn$-A and $NiFe_{0.5}$-A, (e) Tafel plots of $NiFe_{0.1}Sn$-A and $NiFe_1Sn$-A, (f) multipotential steps of $NiFe_{0.5}Sn$-A from 1.47 V to 1.61 V without iR compensation.

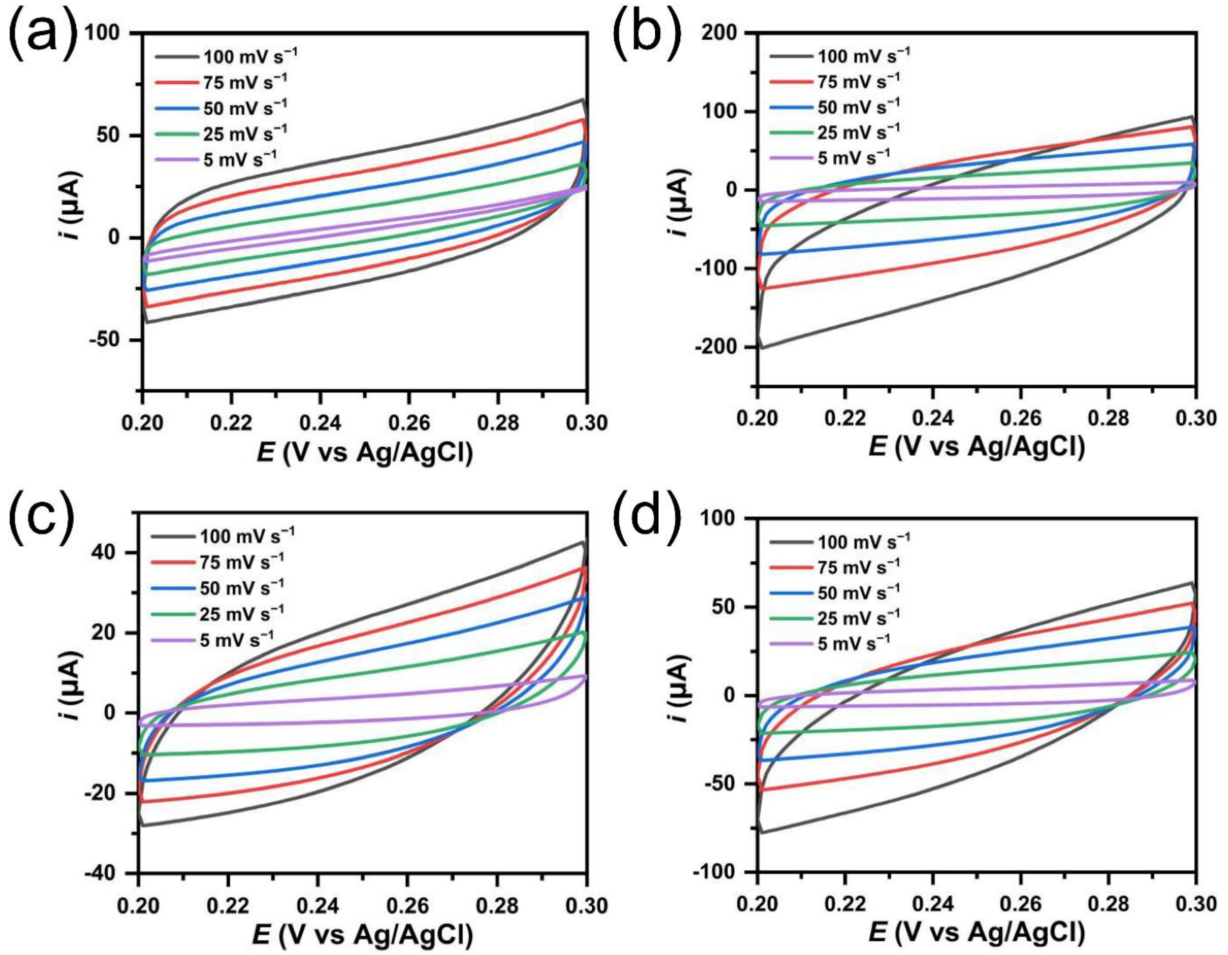

Figure S17. CV curves (a, c) before and (b, d) after 2-h anodization of $NiFe_{0.5}Sn$ and $NiFe_{0.5}$.

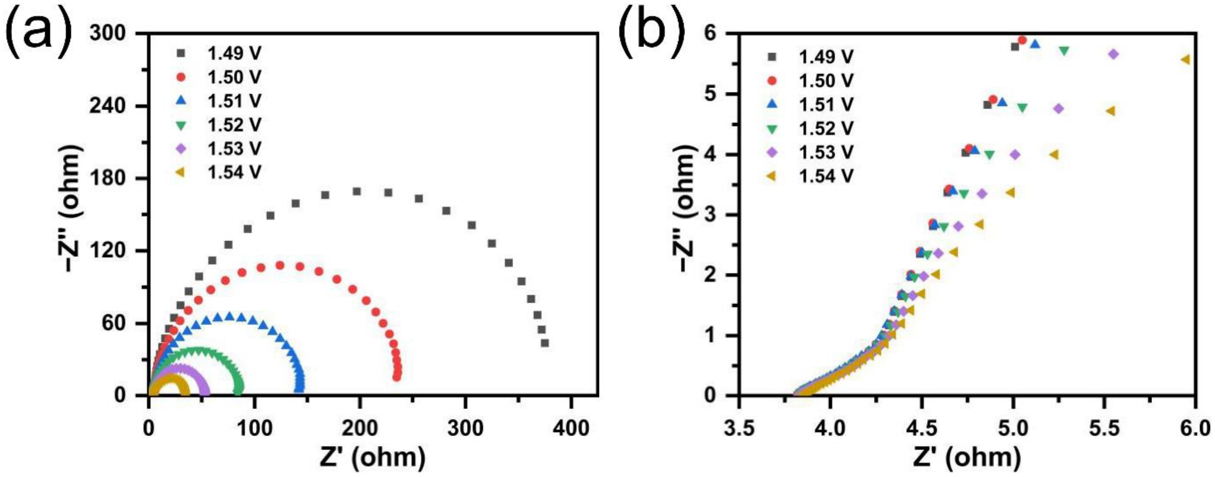

Figure S18. (a) Nyquist plots of NiSn-A at different potentials and (b) the corresponding enlarged views.

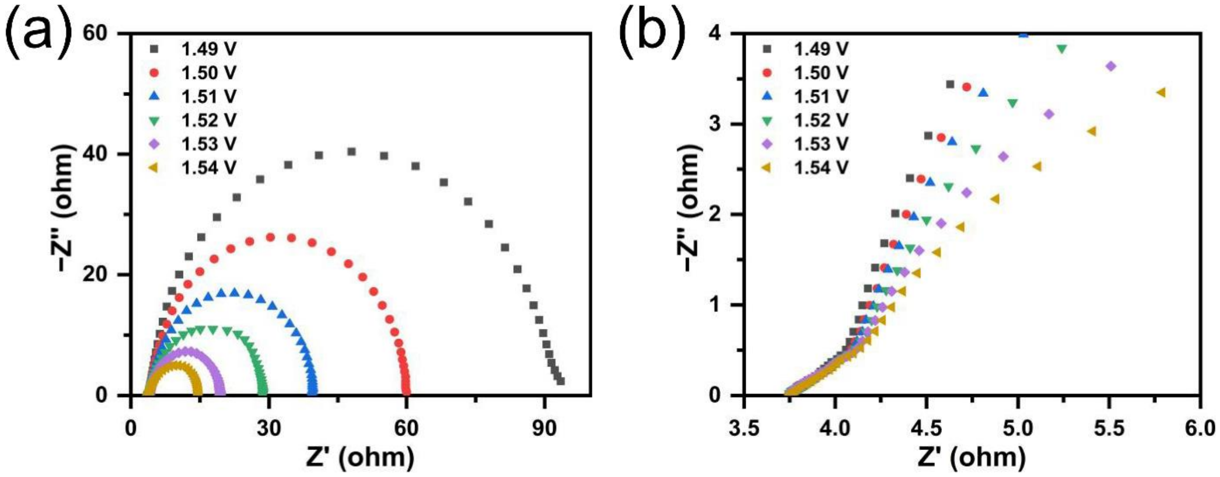

Figure S19. (a) Nyquist plots of $NiFe_{0.5}Sn$-A at different potentials and (b) the corresponding enlarged views.

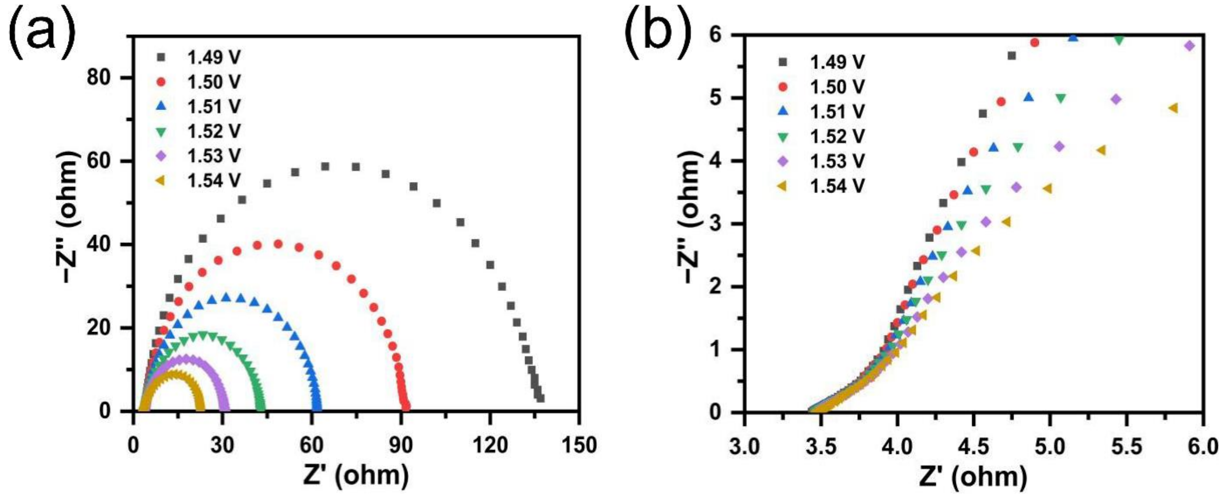

Figure S20. (a) Nyquist plots of $NiFe_{0.5}$-A at different potentials and (b) the corresponding enlarged views.

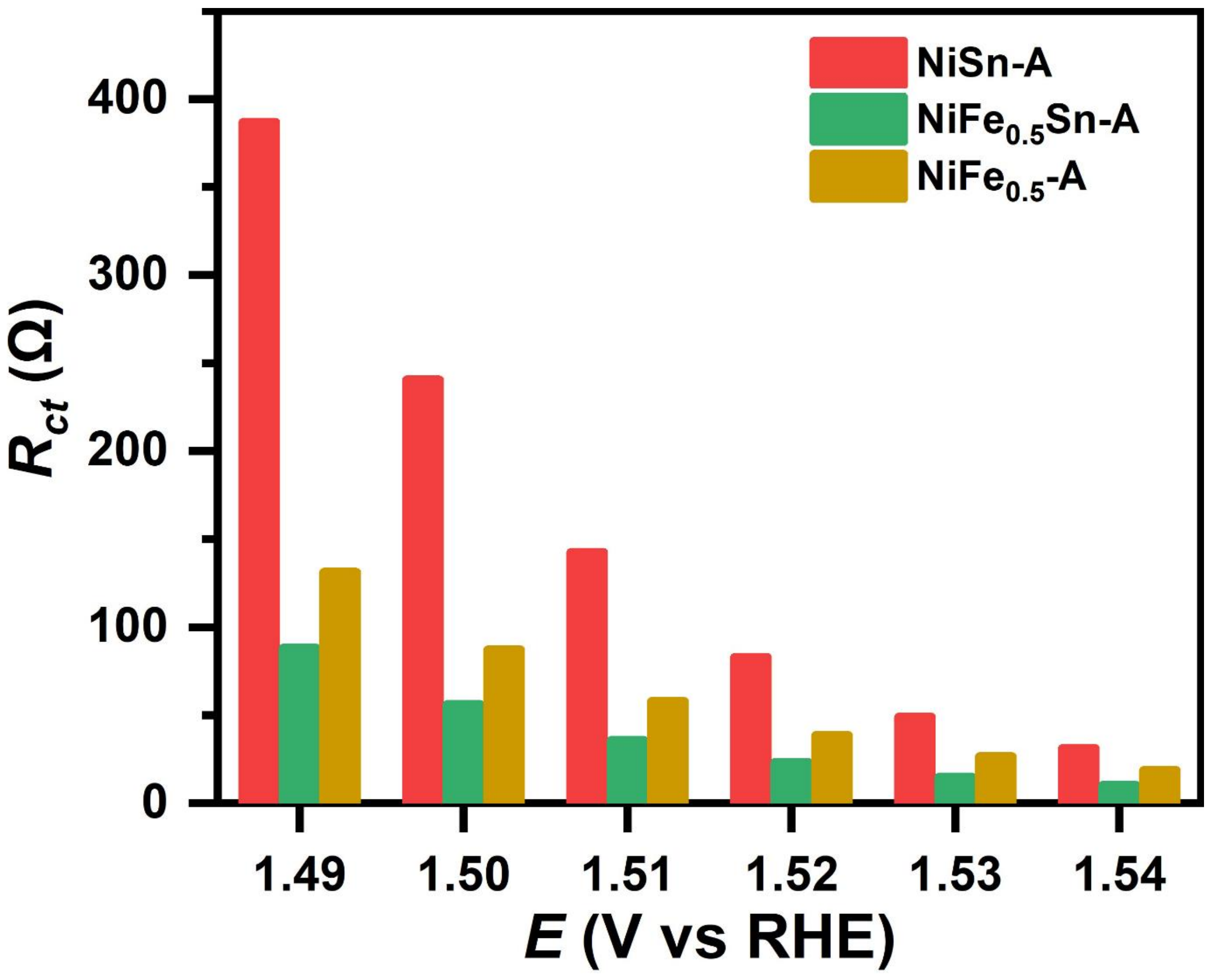

Figure S21. R_{ct} values of NiSn-A, $NiFe_{0.5}Sn$-A and $NiFe_{0.5}$-A at different potentials.

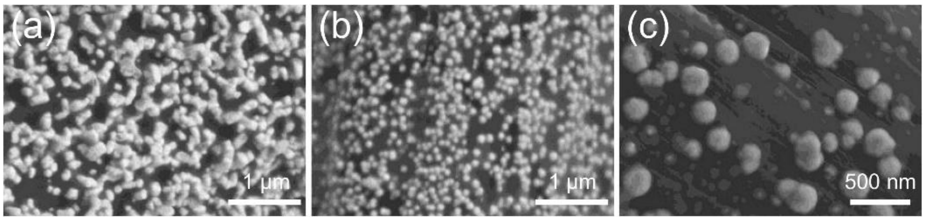

Figure S22. SEM images of NiSn-A, $NiFe_{0.5}Sn$-A and $NiFe_{0.5}$-A.

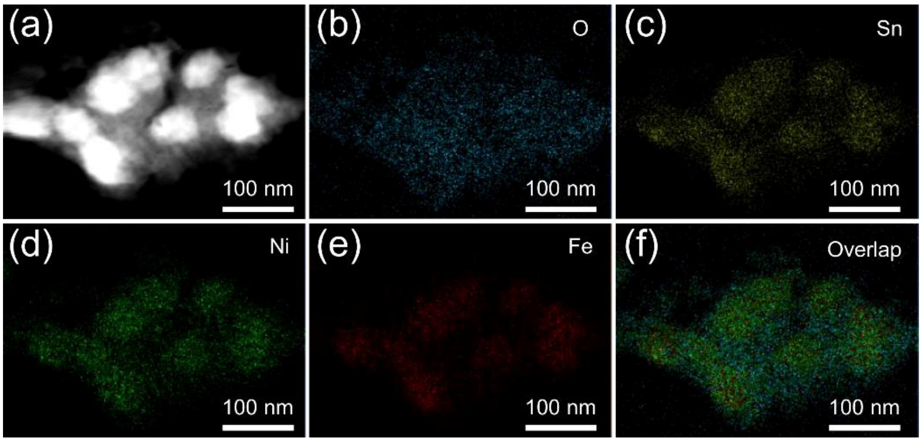

Figure S23. TEM elemental mapping of $NiFe_{0.5}Sn$-A.

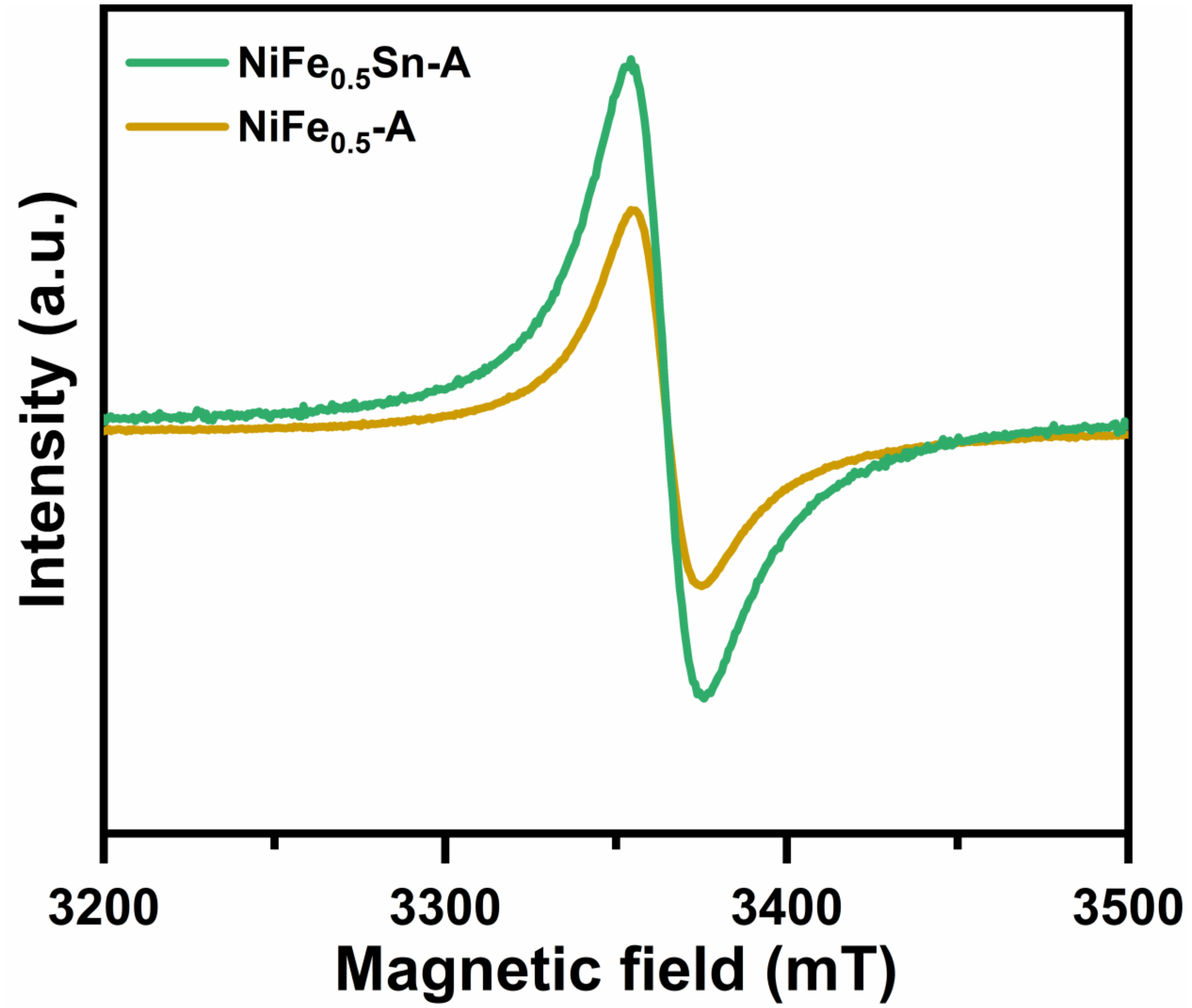

Figure S24. X-band EPR spectra of $NiFe_{0.5}$Sn-A and $NiFe_{0.5}$-A acquired at 100 K (microwave frequency = 9.43 GHz).

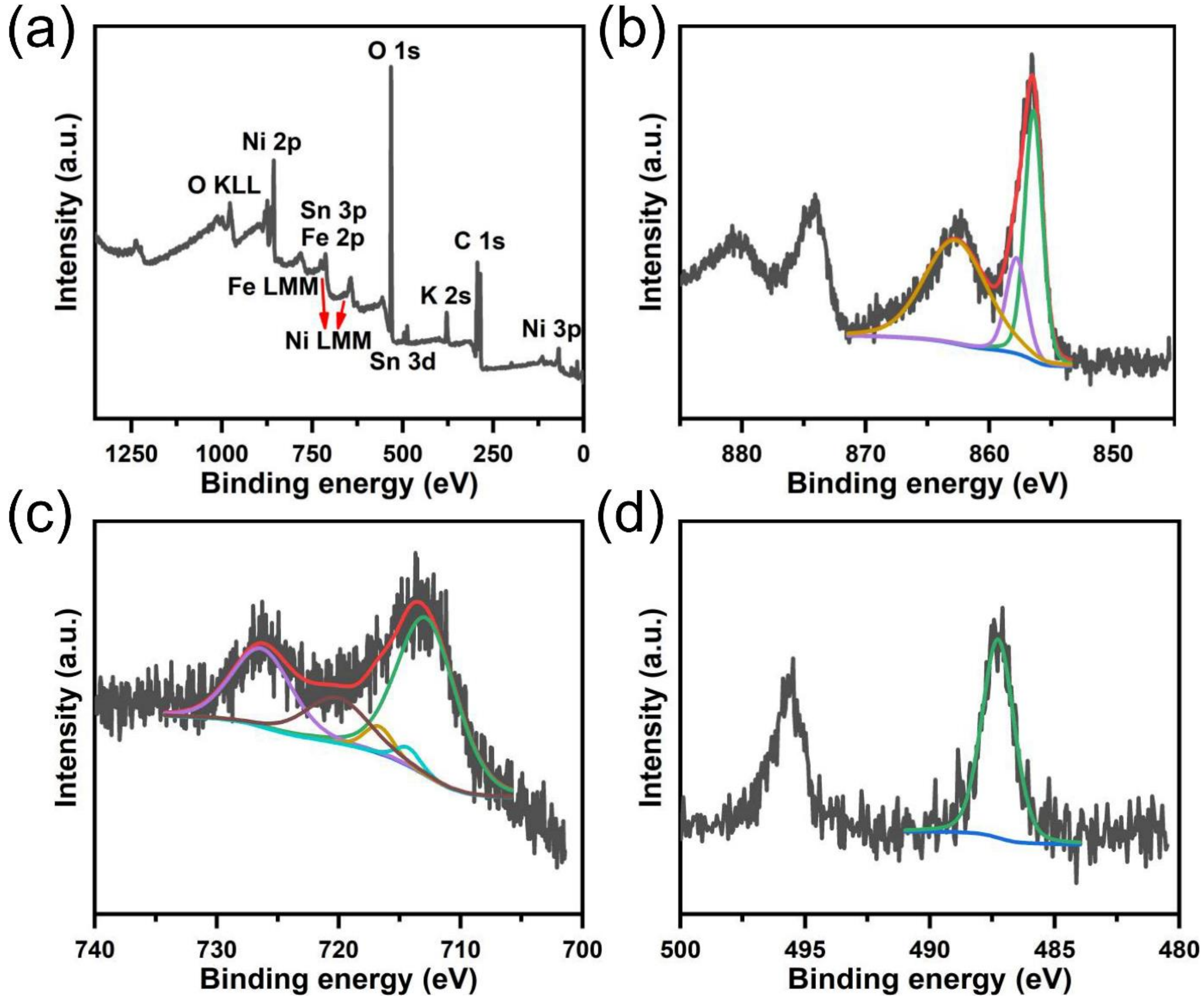

Figure S25. (a) XPS survey, (b) Ni 2p, (c) Fe 2p and (d) Sn 3d XPS spectra of $NiFe_{0.5}Sn$-A.

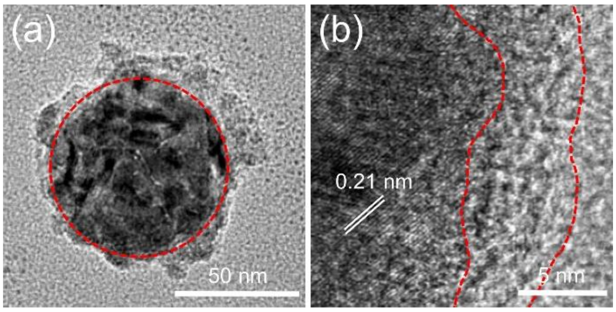

Figure S26. (a) TEM and (b) HRTEM images of $NiFe_{0.5}$-A.

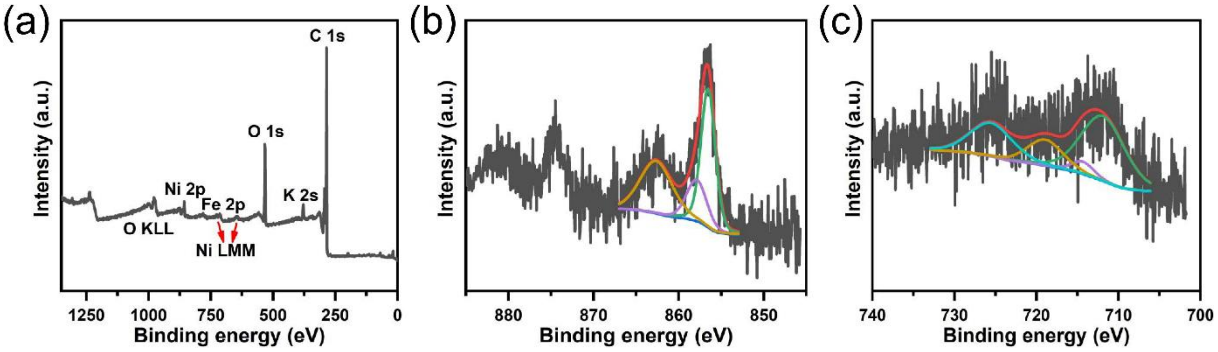

Figure S27. (a) XPS survey, (b) Ni 2p and (c) Fe 2p XPS spectra of $NiFe_{0.5}$-A.

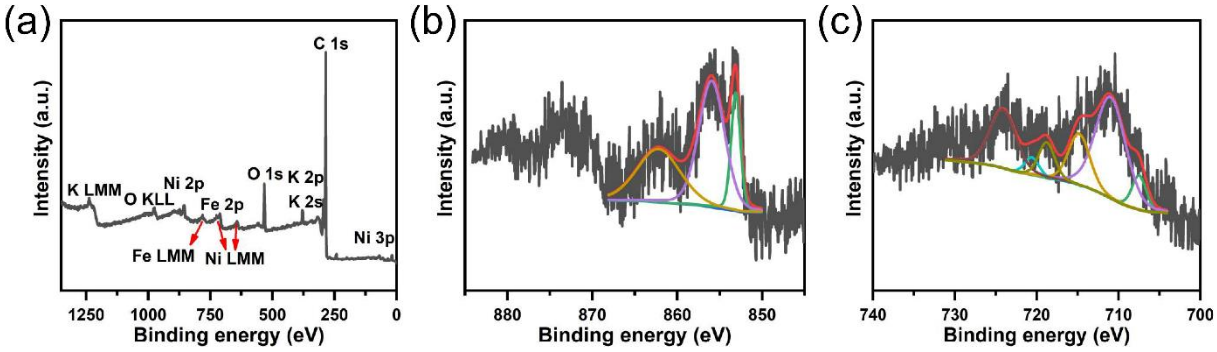

Figure S28. (a) XPS survey, (b) Ni 2p and (c) Fe 2p XPS spectra of $NiFe_{0.5}$-A after Ar^+ ion etching.

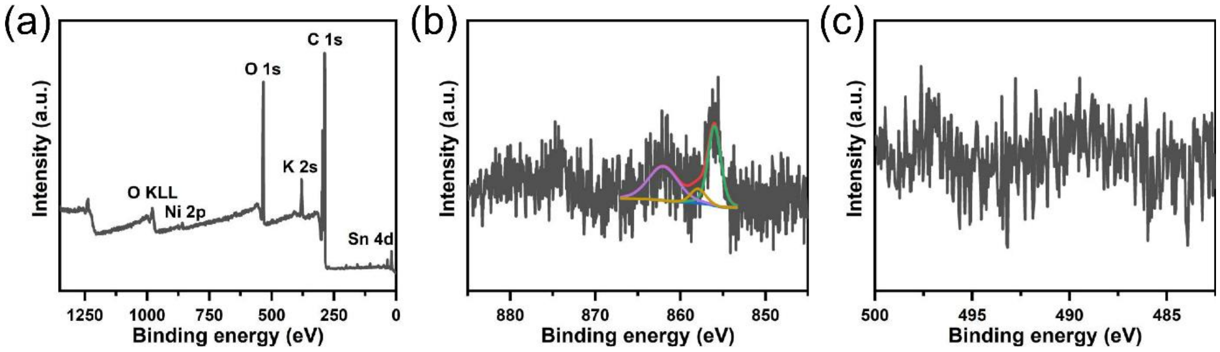

Figure S29. (a) XPS survey, (b) Ni 2p and (c) Sn 3d XPS spectra of NiSn-A.

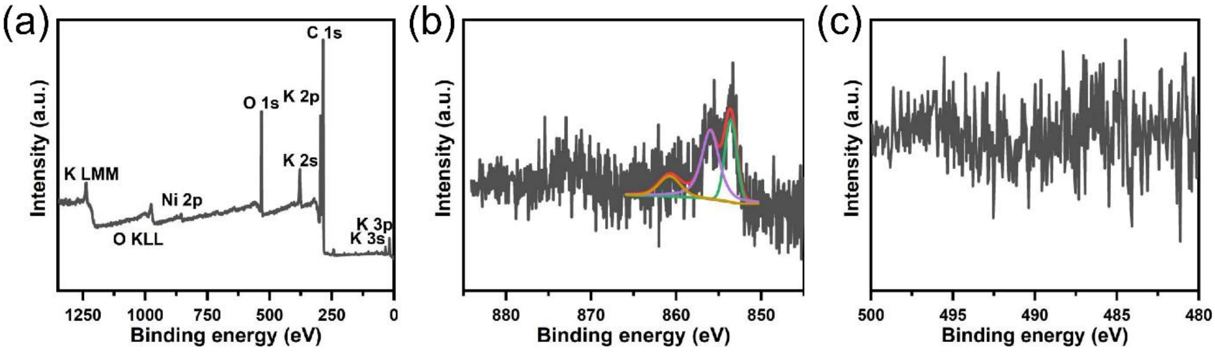

Figure S30. (a) XPS survey, (b) Ni 2p and (c) Sn 3d XPS spectra of NiSn-A after Ar^+ ion etching.

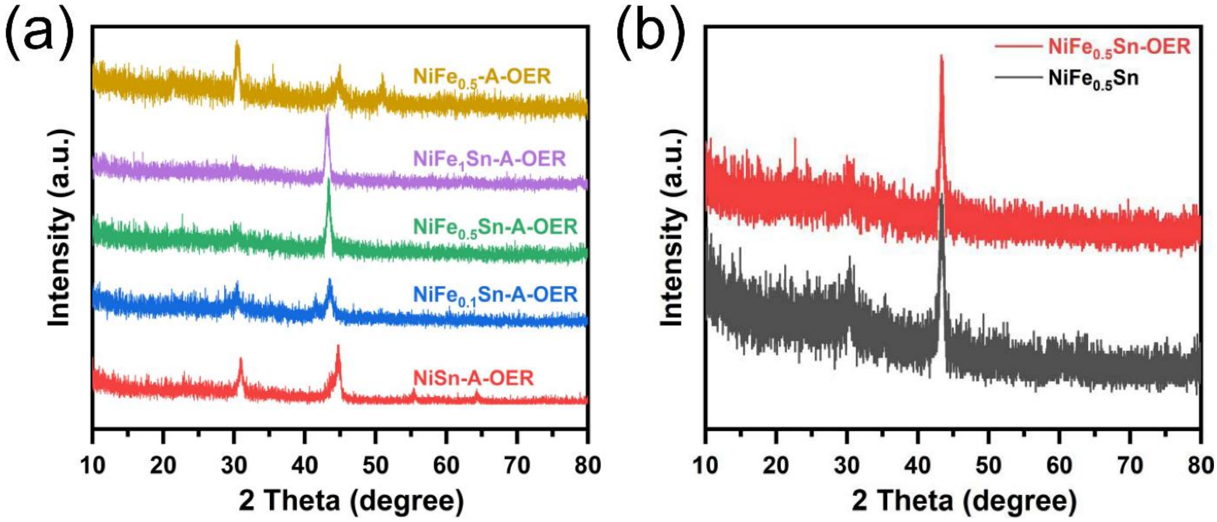

Figure S31. (a) XRD patterns of NiSn-A, $NiFe_xSn$-A, $NiFe_{0.5}$-A after the 40000-s OER duribility test, (b) the comparison of XRD patterns of $NiFe_{0.5}Sn$ and $NiFe_{0.5}Sn$-A after the 40000-s OER duribility test.

Table S1. Comparison of OER performance in alkaline media for $NiFe_{0.5}Sn$-A with other OER elecrocatalysts.

Catalyst	Loading (mg cm^{-2})	Tafel (mV dec^{-1})	Onset overpotential @ 2.5 mA cm^{-2} (mV)	η @ 10 mA cm^{-2} (mV)	Ref.
$NiFe_{0.5}Sn$-A	0.048	50	227	260	This work
NiSn-A	0.019	59	279	310	This work
$NiFe_{0.5}$-A	0.029	52.7	257	287	This work
Exfoliated NiFe LDH	0.07	40	275	302	*Nature Commun.* **2014**, *5*, 4477
15 at% Fe holey film	N/A	38	~250	295	*ACS Catal.* **2017**, *7*, 8406
$Ni_{65}+Fe_{35}(O_xH_y)$	0.025	37	~270	298	*Chem. Commun.* **2019**, *55*, 818
$Na_{0.08}Ni_{0.9}Fe_{0.1}O_2$	0.13	40	~230	260	*Energy Environ. Sci.* **2017**, *10*, 121
NiFeMn-LDH	0.2	47	~230	262	*Chem. Commun.* **2016**, *52*, 908
NiV-LDH	0.143	50	275	320	*Nat. Commun.* **2016**, *7*, 11981
CF-ONFs-O	2.33	69.9	N/A	310	*Adv. Funct. Mater.* **2018**, *28*, 1704177
$Ni_{0.9}Fe_{0.1}$/NC	0.2	45	300	330	*ACS Catal.* **2016**, *6*, 580
nNiFe LDH/NGF	0.25	45	290	337	*Adv. Mater.* **2015**, *27*, 4516
hcp-NiFe@NC	0.25	41	N/A	226	*Angew.Chem.Int. Ed.* **2019**, *58*, 6099
$Ni_{0.75}Fe_{0.25}(OH)_x$	0.35	68	260	310	*Chem. Commun.* **2019**, *55*, 1044

Table S2. The loading of Ni and Fe in NiSn, $NiFe_{0.5}Sn$ and $NiFe_{0.5}$ as determined by ICP-OES.

**Catalyst**	**Ni loading (µg)**	**Fe loading (µg)**
NiSn	4.80	0
$NiFe_{0.5}Sn$	7.89	4.07
$NiFe_{0.5}$	3.71	3.61
